# Dlx1/2 and Otp coordinate the production of hypothalamic GHRH- and AgRP-neurons

**DOI:** 10.1038/s41467-018-04377-4

**Published:** 2018-05-23

**Authors:** Bora Lee, Janghyun Kim, Taekyeong An, Sangsoo Kim, Esha M. Patel, Jacob Raber, Soo-Kyung Lee, Seunghee Lee, Jae W. Lee

**Affiliations:** 1 Center for Neuroscience, Korea Institute of Science and Technology (KIST), Seoul, 02792 Korea; 2 Neuroscience Section, Papé Family Pediatrics Research Center, Department of Pediatrics, Oregon Health and Science University, Portland, OR 97239 USA; 3 Department of Bioinformatics and Life Science, Soongsil University, Seoul, 06978 Korea; 4 Department of Behavioral Neuroscience, Oregon Health and Science University, Portland, OR 97239 USA; 5 Departments of Neurology and Radiation Medicine, and Division of Neuroscience, Oregon National Primate Research Center, Oregon Health and Science University, Portland, OR 97239 USA; 6 Vollum Institute, Oregon Health and Science University, Portland, OR 97239 USA; 7 College of Pharmacy and Research Institute of Pharmaceutical Sciences, Seoul National University, Seoul, 08826 Korea

## Abstract

Despite critical roles of the hypothalamic arcuate neurons in controlling the growth and energy homeostasis, the gene regulatory network directing their development remains unclear. Here we report that the transcription factors Dlx1/2 and Otp coordinate the balanced generation of the two functionally related neurons in the hypothalamic arcuate nucleus, GHRH-neurons promoting the growth and AgRP-neurons controlling the feeding and energy expenditure. *Dlx1/2*-deficient mice show a loss-of-GHRH-neurons and an increase of AgRP-neurons, and consistently develop dwarfism and consume less energy. These results indicate that Dlx1/2 are crucial for specifying the GHRH-neuronal identity and, simultaneously, for suppressing AgRP-neuronal fate. We further show that Otp is required for the generation of AgRP-neurons and that Dlx1/2 repress the expression of Otp by directly binding the *Otp* gene. Together, our study demonstrates that the identity of GHRH- and AgRP-neurons is synchronously specified and segregated by the Dlx1/2-Otp gene regulatory axis.

## Abstract (web summary)

In the hypothalamus, the arcuate nucleus (ARC) contains AgRP-neurons that regulate energy balance as well as GHRH-neurons that regulate linear growth. Here, the authors looked at how the transcription factors Dlx1/2 and Otp link development of AgRP- and GHRH-neurons.

## Introduction

The hypothalamus is a central regulator of the homeostatic processes that are essential to survival and reproducton^1,2^. Among the multiple hypothalamic nuclei, the arcuate nucleus (ARC) is particularly receptive to various peripheral cues because it is located in proximity to the blood stream. Thus, in the central nervous system, the ARC serves as a primary gatekeeper and processor for the peripheral signals in directing the growth, energy balance, and reproductive behaviors in response to such cues^1,2^. Numerous investigations for the past decades^1,2^ significantly enhanced our understanding of physiological roles of the ARC. The highly interconnected actions among the arcuate neurons in striking the body homeostasis raise the possibility that the distinct arcuate neuronal types are generated in a coordinated manner during hypothalamus development. However, the gene regulatory network that orchestrates their production remains poorly understood.

The ARC is composed of many different types of neurons that express specific sets of neuropeptides and elicit disparate physiological actions^3^. These include the agouti-related protein (AgRP)-neurons and pro-opiomelanocortin (POMC)-neurons, which play crucial roles in maintaining energy homeostasis^1^. The AgRP-neurons secrete AgRP and neuropeptide Y (NPY), both of which increase food intake and decrease energy expenditure. In contrast, the POMC-neurons express both cocaine and amphetamine-related transcript (CART) and melanocyte-stimulating hormone-α (αMSH), which is derived from the precursor peptide POMC. The αMSH released from POMC-neurons decreases food intake and increases energy expenditure^1^. Thus, the AgRP- and POMC-neurons exhibit the opposing activities in determining the levels of food intake and energy consumption. Interestingly, 17~25% of AgRP-neurons are derived from POMC-expressing cells^4^, indicating a close developmental link between AgRP- and POMC-neurons. Another critical neuronal type in the ARC is the growth hormone-releasing hormone (GHRH)-neurons that release GHRH^2^. The GHRH triggers the secretion of the growth hormone (GH) from the anterior pituitary gland^2^. GH then triggers expression in the liver of insulin-like growth factor 1 (IGF1). IGF1 regulates bone epiphyses, growth plate development, muscle and adipose tissue development, and glucose homeostasis^2^. The control for nutritional status and linear growth needs to be coupled. Supporting this notion, it has been well-established that GH/GHRH levels are inversely correlated with the circulating level of the anorexic hormone leptin that targets arcuate neurons to control the energy balance^5–7^. Given the physiological importance of the tight linkage between the energy balance and linear growth, it is possible that the production of GHRH-neurons is coordinated with the generation of AgRP- and/or POMC-neurons. To date, however, little has been known about whether and how the development of growth-promoting GHRH-neurons is connected to the formation of AgRP- or POMC-neurons that regulate the energy homeostasis.

Distal-less homeobox-1 (Dlx1) and its homolog Dlx2 are the homeodomain transcription factors that play important and redundant roles in the fate specification of GABA^+^ and tyrosine hydroxylase (TH)^+^ neurons in the forebrain^8–12^. The immunochemical analyses with a pan-Dlx antibody detecting multiple Dlx factors revealed that Dlx factors are expressed in TH^+^ neurons and non-AgRP-neuronal type GABA^+^ neurons in the ARC^13^. Adult Dlx1-null mice show a twofold reduction in the number of TH^+^ neurons in the ARC^13^; whereas, the role of Dlx2 in the hypothalamus remains unexplored. We found that Dlx1 and Dlx2 are robustly expressed in the embryonic ARC by analyzing the three datasets, www.brain-map.org, www.genepaint.org, and the seminal work of Blackshaw and colleagues^14^, consistent with the earlier report^15^. This expression pattern suggests a possible role of Dlx1 and Dlx2 in the fate specification or differentiation of arcuate neurons. Orthopedia (Otp) is the homeodomain transcription factor that is expressed in the several hypothalamic nuclei including the ARC^16^. Otp-null mice die perinatally and lack somatostatin (Sst)^+^ arcuate neurons^16^, suggesting a role of Otp in the ARC development. Interestingly, Otp and Dlx1 are expressed in alternating domains in the embryonic hypothalamus^16–18^, and Dlx1 expression domain is expanded in presumptive paraventricular nuclei (PVN) in Otp-null mice^16^, suggesting an antagonistic relationship between the two transcription factors. However, the role of Dlx1/2 and Otp in the fate specification of arcuate neurons and how Dlx1/2 and Otp are linked in the gene regulatory network that governs the production of distinct arcuate neuronal types remains poorly understood.

Here our studies revealed that Dlx1/2 and Otp are required for the specification of GHRH- and AgRP-neurons, respectively. Furthermore, we found that Dlx1/2 suppress AgRP-neuronal fate by directly binding and repressing the *Otp* gene, uncovering a novel Dlx1/2-Otp gene regulatory axis critical for the segregation of GHRH- and AgRP-neuronal fates. Together, our data provide, for the first time, the evidence that GHRH- and AgRP-neuronal fates are interconnected, and suggest that the Dlx1/2-Otp axis plays a role in producing a balanced ratio of AgRP- to GHRH-neurons in the hypothalamus.

## Results

### Dlx1/2 are expressed in the developing ARC

**Fig. 1:**
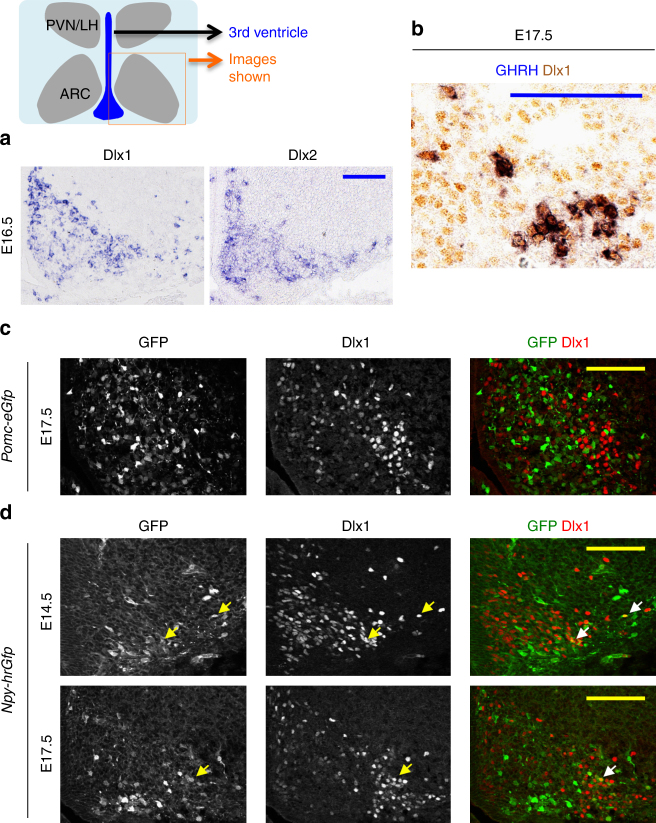
Expression of Dlx1 in the developing ARC. **a**, **b** In situ hybridization (ISH) for Dlx1 and Dlx2 (**a**) and ISH for GHRH combined with immunohistochemistry (IHC) for Dlx1 (**b**). A total of 88.5 ± 5% of GHRH^+^ cells were Dlx1^+^ cells in IHC (**b**). **c**, **d** IHC analyses of Dlx1 expression in *Pomc-eGfp* (**c**) and *Npy-hrGfp* (**d**) embryos using our home-made antibodies against Dlx1. In *Pomc-eGfp*, none of GFP^+^ cells was Dlx1^+^ in IHC (**c**). In E14.5 *Npy-hrGfp*, 15 ± 1% of GFP^+^ cells were Dlx1^+^ in IHC, while 93 ± 1% of Dlx1^+^ cells did not express GFP (**d**). In E17.5 *Npy-hrGfp*, 12 ± 2% of GFP^+^ cells were Dlx1^+^ in IHC, while 90 ± 3% of Dlx1^+^ cells did not express GFP (**d**). Quantification was performed with multiple embryos and at least three sections from each embryo, and representative images for only one side of the ARC are as shown. Scale bars, 100 μm

To understand the role of Dlx1 and Dlx2 in ARC development, we investigated their expression pattern in the developing murine hypothalamus. We found that both Dlx1 and Dlx2 are highly enriched in the ARC at E16.5 (Fig. 1a). Consistently, the publically available datasets (www.brain-map.org, www.genepaint.org and Shimogori et al.^14^) show that Dlx1 and Dlx2 are highly expressed in the mediobasal hypothalamus, from which the ARC is derived, at E11.5, and that Dlx1/2 continue to be expressed in the ARC in the mature brain. These overlapping expression patterns suggest that Dlx1 and Dlx2 may function redundantly in the ARC, similarly to their roles in the developing forebrain^11^.

We next analyzed the expression of Dlx1 in GHRH-, AgRP-, and POMC-neurons, the three major types of ARC neurons that control the growth and energy balance. In the embryonic ARC, Dlx1 was expressed in most GHRH-neurons (Fig. 1b), whereas it was not expressed in POMC-neurons that are visualized by the *Pomc-eGfp* transgenic allele^19^ (Fig. 1c). As AgRP expression in the ARC begins only perinatally, we labeled AgRP-neurons in the embryonic ARC using the *Npy-hrGfp* transgenic mice^20^, in which GFP is expressed in embryonic AgRP-neurons before the induction of AgRP. At E14.5 and E17.5, Dlx1 was mostly excluded from GFP^+^ neurons (Fig. 1d), although 12~15% of GFP^+^ neurons that express GFP at a low level were co-stained with Dlx1 (arrows in Fig. 1d).

We also examined the expression of Dlx1 in other cell types in the ARC. Our results revelaed that Dlx1 is expressed neither in KNDy-neurons (which express Kiss1) (Supplementary Fig. 1a, b) nor tanyctes in the ARC (Supplementary Fig. 1c). We also found that Dlx1 is expressed in neither Olig2^+^ oligodendrocytes nor GFAP + astrocytes in the ARC (Supplementary Fig. 2a, b).

Taken together, these results suggest that Dlx1 may play a role in the development of GHRH-neurons, but less likely in the formation of POMC-neurons, AgRP-neurons or other arcuate cell types.

### Dual roles of Dlx1/2 in GHRH-/AgRP-neuronal fate decision

**Fig. 2:**
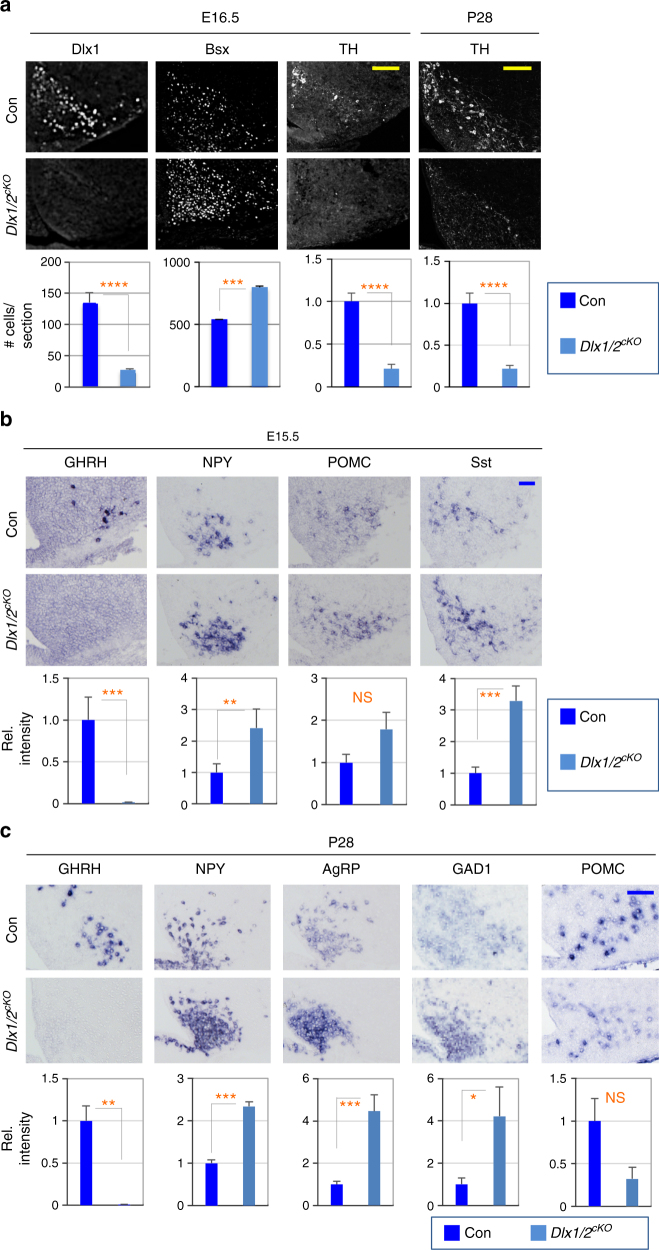
Expression of arcuate neuropeptides in *Dlx1/2*^*cKO*^ mice. **a** IHC analyses of E16.5 *Dlx1/2*^*cKO*^ embryos (*n* = 4) revealed a significant decrease in the number of Dlx1^+^ and TH^+^ cells but a significant increase in the number of Bsx^+^ cells relative to their littermate controls (*n* = 4). Similar observations were made for TH in the ARC of P28 *Dlx1/2*^*cKO*^ mice (*n* = 3) relative to their controls (*n* = 3). **b** ISH analyses of *Dlx1/2*^*cKO*^ embryos (*n* = 4) revealed abolished expression of GHRH but a significant increase in the expression of NPY and Sst relative to their littermate controls (*n* = 4). **c** In ISH analyses, P28 *Dlx1/2*^*cKO*^ mice (*n* = 3) showed a significant increase in the expression of NPY, AgRP and GAD1 while losing the expressing of GHRH in comparison to their control mice (*n* = 3). The expression of POMC was not significantly altered (**b**, **c**). Quantification was performed with multiple embryos as indicated and at least 3 sections from each embryo, and representative images for only one side of the ARC are as shown. Scale bars, 100 μm. Statistical differences were determined by Student’s *t*-test: ∗*p* < 0.05, ∗∗*p* < 0.01, ∗∗∗*p* < 0.001, and ∗∗∗∗*p* < 0.0001. Bars represent mean, error bars indicate the SEM

To inactivate both Dlx1 and Dlx2 in the developing ARC, we generated conditional Dlx1/2-null mice (*Dlx1/2*^*cKO*^) in which the genomic region encompassing *Dlx1* and *Dlx2* genes is deleted by *Nkx2.1-Cre*, which broadly deletes genes in several cell types of the developing ARC (e.g., GABA^+^, NPY^+^, POMC^+^, TH^+^ neurons)^21,22^. Our immunostaining analysis confirmed that Dlx1 expression was eliminated in the ARC of *Dlx1/2*^*cKO*^ mice (Fig. 2a, Supplementary Fig. 3a). The overall shape of ARC was comparable between *Dlx1/2*^*cKO*^ embryos and their controls. Interesingly, the size of the ARC of *Dlx1/2*^*cKO*^ mice became smaller than their controls at postnatal stages, but the numer of NeuN^+^ neurons were not different (Supplementary Fig. 3b), suggesting that the ARC of *Dlx1/2*^*cKO*^ mice may have a non-neuronal issue such as a decrease in extracellular matrix. Importantly, GHRH-neurons were lost in the ARC of *Dlx1/2*^*cKO*^ mice at E15.5 and P28 (Fig. 2b,c). TH^+^ neurons were also significantly reduced in the ARC of *Dlx1/2*^*cKO*^ mice (Fig. 2a). Given that ~76% of GHRH-neurons express TH^23^, a loss-of-GHRH-neurons in *Dlx1/2*^*cKO*^ mice likely contributes to the reduction of TH^+^ neurons. In addition, the expression of Gsx1 and Hmx2, which drive the expression of GHRH and Gsx1, respectively^24,25^, was significantly reduced in E15.5 *Dlx1/2*^*cKO*^ ARC in comparison to their controls (Supplementary Fig. 3c). These results indicate that Dlx1/2 are required for the production of GHRH-neurons during hypothalamus development.

Next, we asked whether the removal of Dlx1/2 affects other ARC neuronal types. Strikingly, we observed a marked increase in NPY expression in the ARC of *Dlx1/2*^*cKO*^ mice at E15.5 (Fig. 2b). Although it was difficult to count the exact number of NPY^+^ cells, more cells were labeled with NPY in *Dlx1/2*^*cKO*^ mice (Fig. 2b). These results raise an interesting possibility that the generation of AgRP-neurons increase in the absence of Dlx1/2. Consistent with this idea, the number of neurons expressing Bsx1, the transcription factor critical for AgRP gene induction^26^, was also increased in the ARC of *Dlx1/2*^*cKO*^ mice (Fig. 2a). Also more cells (as determined by enhanced ISH signal density as well as a larger area of ISH signals) appear to be labeled with Sst in the ARC of *Dlx1/2*^*cKO*^ mice (Fig. 2b). Considering that a subtype of AgRP-neurons express Sst^3^, increased Sst^+^ neurons together with the increase in the number of Bsx^+^ neurons (Fig. 2a) can be, at least partly, attributed to an increase in the number of AgRP-neurons. In the postnatal hypothalamus, similar observations were also made for NPY and AgRP in *Dlx1/2*^*cKO*^ mice (Fig. 2c). Consistent with the report that most AgRP-neurons are GABAergic^27^, we also observed an increased expression of glutamate decarboxylase 1 (GAD1), a GABAergic neuronal marker (Fig. 2c). In contrast to NPY/AgRP, POMC expression did not show a significant difference between *Dlx1/2*^*cKO*^ and control mice (Fig. 2b, c). Together, in the absence of Dlx1/2, the formation of AgRP-neurons is likely facilitated and, interestingly, these aberrantly generated AgRP-neurons persist at postnatal stages in *Dlx1/2*^*cKO*^ mice.

Our results also revealed that the expression of Kiss1, a marker for KNDy-neurons in the ARC, was not significantly altered in *Dlx1/2*^*cKO*^ mice (Supplementary Fig. 4a), consistent with the lack of Dlx1 expression in KNDy-neurons (Supplementary Fig. 1a, b).

Taken together, our results suggest that Dlx1/2 are needed for the generation of GHRH-neurons and the suppression of AgRP-neuronal fate in the developing ARC. Our data suggest that Dlx1/2 play a role in striking a balance between the formation of GHRH- and AgRP-neurons.

### Defects in growth and energy expenditure in Dlx1/2-null mice

**Fig. 3:**
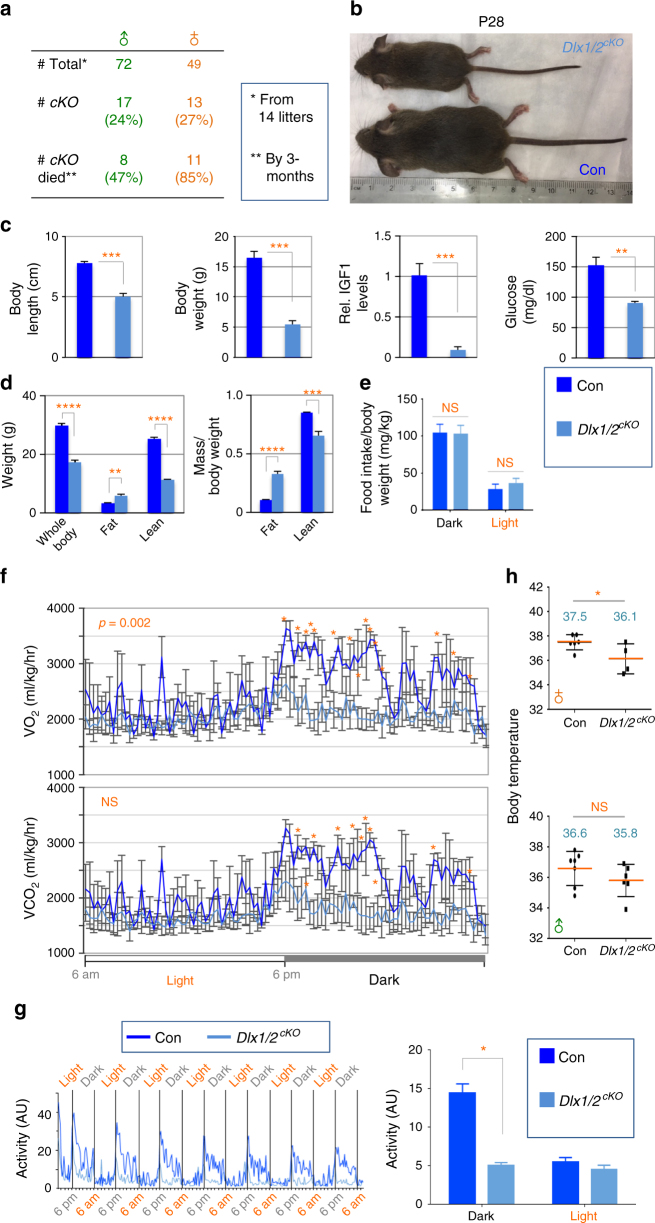
Growth and energy expenditure phenotypes of adult *Dlx1/2*^*cKO*^ mice. **a** Postnatal death of *Dlx1/2*^*cKO*^ mice from a total of 14 litters. **b**, **c** Dwarfism of *Dlx1/2*^*cKO*^ male mice (*n* = 3) (**b**) and qualification of their linear body length, body weight, IGF1 expression in the liver (by qRT-PCR) and serum glucose levels (**c**), relative to their littermate males controls (*n* = 5). The ruler unit in **b** is in cm. **d** MRI analyses of fat and lean mass revealed an increased fat deposition in *Dlx1/2*^*cKO*^ male mice (*n* = 5) relative to their control male mice (*n* = 5). **e** Food intake measurement revealed no significant difference between control (*n* = 6) and *Dlx1/2*^*cKO*^ male mice (*n* = 4) throughout the diurnal cycles. **f** VO_2_/VCO_2_ measurements uncovered a significant decrease in energy expenditure in *Dlx1/2*^*cKO*^ male mice (*n* = 3) relative to their controls (*n* = 3). **g** Circadian home cage activity was continuously measured using a home cage sensor system and mLog software (BioBServe, Germany). **h** Basal body temperature measurement revealed a significant reduction in female *Dlx1/2*^*cKO*^ mice (*n* = 4) relative to their littermate female controls (*n* = 6), as well as a trend for reduced body temperature with male *Dlx1/2*^*cKO*^ mice (*n* = 6) relative to their littermate male controls (*n* = 6), which did not reach statistical significance (*p*, 0.06). The *p*-values by two-way ANOVA test were 0.002 for VO_2_ measurement but not significant (NS) for VCO_2_ measurement, as indicated (**f**). Student’s *t*-test results are as indicated: ∗*p* < 0.05, ∗∗*p* < 0.01, ∗∗∗*p* < 0.001, and ∗∗∗∗*p* < 0.0001 (**c**, **d**, **f**, **h**). The home cage activity and food intake during the light and dark periods were analyzed separately using repeated-measures ANOVA with genotype as the between-subjects factor (**e**, **g**). Bars represent mean, error bars represent the SEM

A remarkable change in GHRH- and AgRP-neurons in *Dlx1/2*^*cKO*^ mice prompted us to ask how this alteration in ARC neuronal composition affects the growth and energy balance in mutant mice. While *Dlx1/2*^*cKO*^ mice were born according to the Mendelian ratio, ~47% of male and ~85% of female *Dlx1/2*^*cKO*^ mice died within 3 months after birth (Fig. 3a), indicating that they are vulnerable to death. Notably, the surviving *Dlx1/2*^*cKO*^ mice were dwarf as evident from their shorter body length and smaller body weight than the littermate controls at P28 (Fig. 3b, c). To investigate the activity of ‘hypothalamic GHRH → pituitary GH → hepatic IGF1’ axis, we monitored the expression of IGF1 in the liver and blood glucose levels. *Dlx1/2*^*cKO*^ mice displayed a drastic decrease in hepatic IGF1, as well as significantly reduced blood glucose levels compared to their littermate controls (Fig. 3c). Our data suggest that the hypothalamic GHRH signaling is likely impaired in *Dlx1/2*^*cKO*^ mice.

The two major actions of AgRP-neurons are to increase food intake and to reduce energy expenditure^1^. Thus we analyzed if *Dlx1/2*^*cKO*^ mice, which appear to possess more AgRP-neurons in the hypothalamus, show any changes in these aspects. Intriguingly, by 3~4 months of age, *Dlx1/2*^*cKO*^ mice became obese and displayed significantly higher fat mass than the littermate controls despite their smaller body weight (Fig. 3d). This accumulated fat mass in *Dlx1/2*^*cKO*^ mice were even more evident when the reduced body weight was taken into consideration (right panel in Fig. 3d). In contrast, the lean mass decreased in *Dlx1/2*^*cKO*^ mice compared to their control mice (Fig. 3d). This obesity phenotype, along with increased AgRP expression in the hypothalamus, suggests that *Dlx1/2*^*cKO*^ mice may take more food and/or comsume less energy. However, *Dlx1/2*^*cKO*^ and their control mice did not significantly differ in food intake throughout the diurnal cycles (Fig. 3e), suggesting that the orexigenic aspect of AgRP-neuronal action was not augmented in *Dlx1/2*^*cKO*^ mice (see discussion). We next monitored the energy expenditure by measuring oxygen consumption and carbon dioxide production. *Dlx1/2*^*cKO*^ mice showed a significantly lower energy consumption than their littermate controls (Fig. 3f). These results suggest that the energy saving aspect of AgRP-neuronal action was enhanced in *Dlx1/2*^*cKO*^ mice and that this at least partly contributed to the obese phenotype. Two of the major mechanisms for AgRP neurons to suppress energy consumption is to block locomotor activity^28^ and thermogenesis^29–31^. Interestingly, *Dlx1/2*^*cKO*^ mice showed strikingly reduced overall activity (Fig. 3g). Moreover *Dlx1/2*^*cKO*^ mice became sicker and died even earlier when housed individually than when accommodated as a group, pointing to the possibility that *Dlx1/2*^*cKO*^ mice are impaired in maintaining the core body temperature. Indeed, *Dlx1/2*^*cKO*^ female mice showed a significantly lower body temperature than their controls at room temperature, and *Dlx1/2*^*cKO*^ male mice also displayed a similar trend for lower body temperature without reaching statistical significance (Fig. 3h). Also, the body temperature of *Dlx1/2*^*cKO*^ female mice dropped more quickly than their controls upon the cold exposure (5 °C) (Supplementary Fig. 3d). When we measured the expression of Ucp1, the major thermogenic factor in the brown adipocytes, 60 min after the cold exposure, when the body temperature of *Dlx1/2*^*cKO*^ female mice was lowest (Supplementary Fig. 3d), Ucp1 expression was significantly lower in *Dlx1/2*^*cKO*^ female mice relative to control female mice (Supplementary Fig. 3e). Moreover, under the same condition (60 min post cold exposure), brown adipocytes of *Dlx1/2*^*cKO*^ female mice showed hypertrophy in comparison to control female mice (Supplementary Fig. 3f). Together, *Dlx1/2*^*cKO*^ mice, which appear to possess an increased number of AgRP-neurons in the hypothalamus, showed suppressed energy consumption but not increased food intake.

Collectively, our analyses revealed that *Dlx1/2*^*cKO*^ mice exhibit physiological changes in growth and energy balance in agreement with a loss-of-GHRH-neurons and a gain-of-AgRP-neurons in the hypothalamus.

### Dlx1/2 directly bind and repress the *Otp* gene

**Fig. 4:**
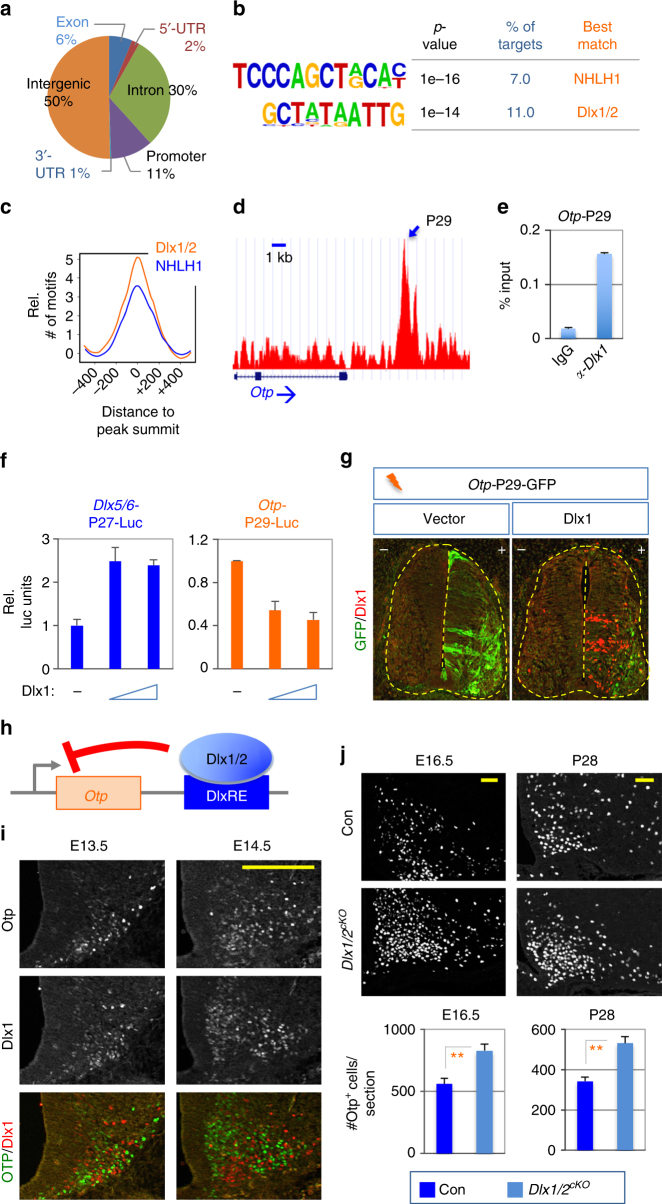
Identification of *Otp* as a direct target gene of Dlx1/2. **a** Location of Dlx1 ChIPseq peaks in the genome. **b**, **c** De novo motif analyses revealed that binding sites for two transcription factors, NHLH1 and Dlx1/2, are enriched in our Dlx1 ChIPseq peaks (**b**), which are mostly located in the summit area of Dlx1 ChIPseq peaks (**c**). **d**, **e** Identification of Dlx1 ChIPseq peak P29 associated with *Otp* (**d**) and validation of Dlx1 binding to this peak in ChIP using anti-Dlx1 antibody and E16.5 hypothalami (**e**). Bars represent mean, error bars indicate the SD (**e**). **f** Luciferase assays in HEK293 cells, in which Dlx1 activates the activity of *Dlx5/6*-P27-Luc reporter and represses the activity of *Otp*-P29-Luc reporter in a dose dependent manner (5 and 10 ng of Dlx1 expression vector). Bars represent mean, error bars indicate the SD. The reporter assays were repeated four times, which produced similar results. A representative set of results is as shown. **g** *In ovo* expression of GFP by a reporter directed by P29 is strongly suppressed by coexpressed Dlx1. **h** Schematic model for Dlx1/2 to suppress Otp expression via direct binding of *Otp*-DlxRE by Dlx1/2. **i** IHC analyses of Otp/Dlx1 expression in E13.5 and E14.5 embryos using our home-made antibodies against Otp and Dlx1, in which Dlx1 and Otp were expressed in a mutually exclusive pattern. **j** In IHC analyses, the number of Otp^+^ cells was significantly increased in *Dlx1/2*^*cKO*^ mice (*n* = 4 for E16.5, *n* = 3 for P28) relative to their littermate controls (*n* = 4 for E16.5, *n* = 3 for P28). In both stages, *p*-values in Student’s *t*-test were <0.01. Quantification was performed with multiple embryos as indicated and at least three sections from each embryo, and representative images for only one side of the ARC are as shown. Scale bars, 100 μm. Bars represent mean, error bars represent the SEM

To elucidate the molecular mechanism by which Dlx1/2 control the formation of GHRH- and AgRP-neurons in the developing hypothalamus, we performed ChIPseq analyses with our anti-Dlx1 antibody (Supplementary Fig. 5) in E16 mouse hypothalami. We obtained a total of 932 Dlx1-bound ChIPseq peaks (*p* < 0.001), including a peak (P27) encompassing the previously reported Dlx1/2-bound binding site^32^ in the intergenic region of *Dlx5* and *Dlx6* (Supplementary Fig. 6a, Supplementary Data 1, GSE104372). Interestingly, ~80% of Dlx1-bound ChIPseq peaks were located in intergenic or intronic regions, whereas only ~11% of the peaks were found in the promoter region (Fig. 4a). De novo motif analysis revealed that two motifs are significantly enriched in Dlx1-bound genomic regions; Dlx1/2- and NHLH1-binding motifs (Fig. 4b). Both motifs were located around the summit of Dlx1-bound peaks (Fig. 4c), suggesting that they serve as the sequences recruiting Dlx1 to the motif-bearing genomic regions.

Among putative direct target genes of Dlx1 that our ChIPseq analyses identified, *Otp* is a good candidate for mediating the action of Dlx1 in the ARC, given that Otp is expressed in the ARC and it is important for the formation of Sst^+^ neurons in this region^16^. Dlx1 was recruited to a peak located at ~4 kb downstream region of *Otp*, named *Otp*-P29 (Fig. 4d). Our ChIP assays in E16.5 hypothalami further validated the binding of Dlx1 to *Otp*-P29 (Fig. 4e). *Otp*-P29 contains multiple Dlx1/2-binding motifs that are evolutionarily conserved, but not NHLH1-binding motifs (Supplementary Fig. 6b). To test how Dlx1/2 influence the transcriptional activity of *Otp*-P29, we constructed a luciferase reporter whose luciferase expression is directed by *Otp*-P29. We also generated another luciferase reporter containing the known Dlx1-binding site in the Dlx5/6 intergenic region^32^ (*Dlx5/6*-P27, Supplementary Fig. 6a). Dlx1 and Dlx2 enhanced the transcriptional activity of *Dlx5/6*-P27 in luciferase assays in HEK293 cells; whereas, the DNA-binding-defective mutant form of Dlx2 (Dlx2-QE, Supplementary Fig. 5a) failed to do so (Fig. 4f, Supplementary Fig. 7a), consistent with the report that Dlx1/2 activate the expression of Dlx5/6 by binding to *Dlx5/6*-P27 region^32^. In contrast to *Dlx5/6*-P27, Dlx1 and Dlx2 repressed the transcriptional activity of *Otp*-P29, whereas Dlx2-QE did not (Fig. 4f, Supplementary Fig. 7a), suggesting that Dlx1/2 directly bind to Dlx1/2-response elements and inhibit the transcriptional activity of *Otp*-P29. To further test the effect of Dlx1 on *Otp*-P29 in vivo, we generated a GFP reporter whose GFP expression is driven by *Otp*-P29, and electroporated this *Otp*-P29:GFP reporter with Dlx1 expression vector or control vector in chick neural tube using an *in ovo* electroporation technique. Dlx1 inhibited GFP expression driven by *Otp*-P29 in the neural tube (Fig. 4g), indicating that Dlx1 binding to *Otp*-P29 leads to transcriptional repression in vivo (Fig. 4h). Interestingly, the ectopically expressed Dlx1 also suppressed the endogenous expression of Otp, but not that of Isl1, in the ventral neural tube (Supplementary Fig. 7b), further demonstrating that Dlx1 inhibits Otp expression in vivo.

To test the regulatory relationship between Dlx1/2 and Otp in the ARC, we examined their expression profile in the developing ARC. Dlx1 and Otp were expressed in a mutually exclusive pattern in the ARC of E13.5/14.5 WT mice (Fig. 4i). Interestingly, Otp^+^ neurons markedly increased in the ARC of *Dlx1/2*^*cKO*^ mice at E13.5-P28 (Supplementary Fig. 3a, Fig. 4j), further supporting the idea that Dlx1/2 suppresses Otp expression.

Taken together, our data demonstrate that Dlx1/2 bind their binding sites located at ~4 kb downstream of the *Otp* gene and inhibit the expression of Otp in the developing ARC.

### Otp is critical for the formation of AgRP-neurons

**Fig. 5:**
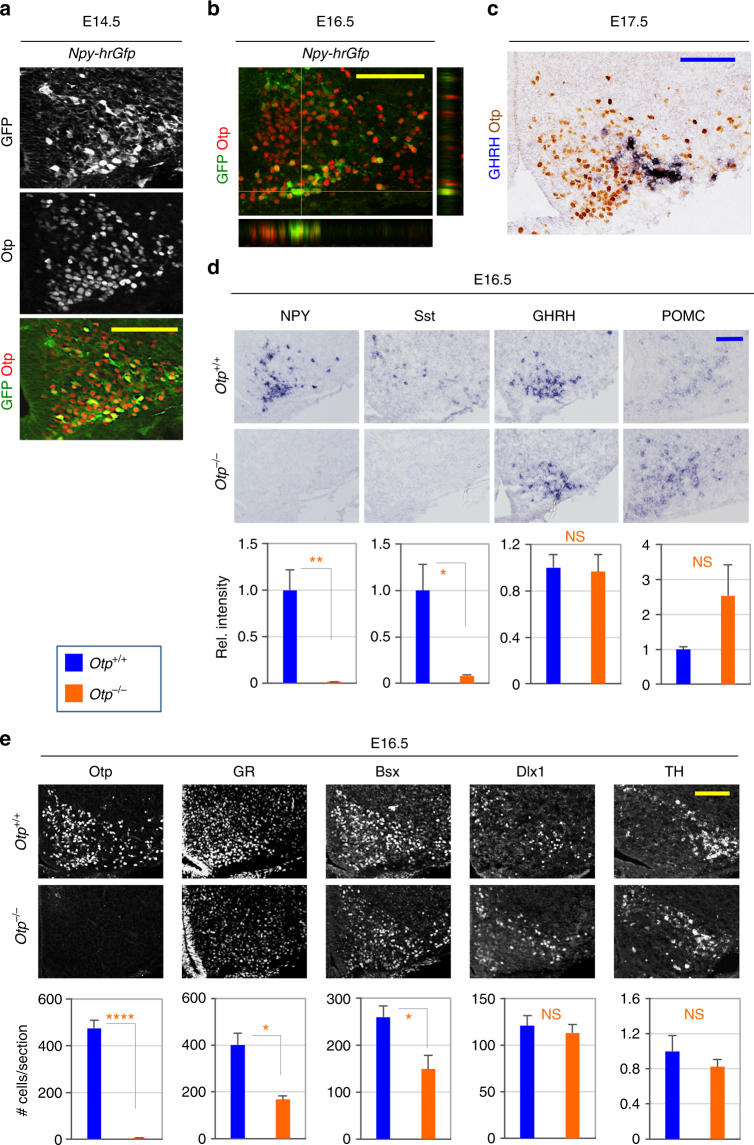
Otp expression in AgRP-neurons and expression of arcuate neuropeptides in Otp-null embryos. **a** IHC analyses of Otp expression in *Npy-hrGfp* embryos using our home-made antibodies against Otp. 86 ± 3% of GFP^+^ cells were Otp^+^ cells in IHC. **b** A confocal Z-stack analysis was also performed with IHC analyses of Otp expression in E16.6 *Npy-hrGfp* embryos. **c** ISH for GHRH combined with IHC for Otp. 3 ± 1% of GHRH^+^ cells were Otp^+^ cells in IHC. **d** ISH analyses of E16.5 Otp-null embryos (*n* = 3) revealed a significant decrease in the expression of NPY and Sst but neither GHRH nor POMC in comparison to their littermate controls (*n* = 4). **e** IHC analyses of E16.5 Otp-null embryos (*n* = 4) revealed a significant decrease in the number of Otp^+^, GR^+^, and Bsx^+^ cells but neither Dlx1^+^ or TH^+^ cells relative to their littermate controls (*n* = 4). Quantification was performed with multiple embryos as indicated and at least three sections from each embryo, and representative images for only one side of the ARC are as shown. Scale bars, 100 μm. Student’s *t*-test results are as indicated: ∗*p* < 0.05, ∗∗*p* < 0.01, and ∗∗∗∗*p* < 0.0001 (**d**, **e**). Bars represent mean, error bars represent the SEM

The identification of *Otp* as a downstream target gene of Dlx1/2 in the hypothalamus led us to ask if Otp plays any role in the development of GHRH- and AgRP-neurons for which Dlx1/2 play important roles. In the embryonic ARC of *Npy-hrGfp* mice, Otp was expressed in most GFP^+^ neurons (Fig. 5a, b). In contrast, most GHRH-neurons did not express Otp (Fig. 5c). We also found that KNDy-neurons, tanycytes, oligodendrocytes, astrocytes and TH^+^ neurons in the ARC do not express Otp (Supplementary Fig. 1a, c; Supplementary Fig. 2, a-c). These results suggest a role for Otp specifically in AgRP-neuronal development. Indeed, NPY^+^, AgRP^+^, and Sst^+^ neurons were entirely lost in the ARC of Otp-null mice at E16.5 and E18.5 (Fig. 5d, Supplementary Fig. 4b). Also Gad1 ISH intensity was significantly reduced in Otp-null mice at P0 (Supplementary Fig. 4a), consistent with the loss-of-AgRP-neurons that are mostly GABAergic^27^. These results suggest that Otp is required for the generation of AgRP-neurons. Consistently, the neurons that express GR and Bsx, the two transcription factors that are important for the expression of AgRP and NPY in the ARC^26,33^, were greatly reduced in the Otp-deficient mice (Fig. 5e). In contrast, POMC^+^ and GHRH^+^ (as determined by ISH signal intensity) and TH^+^ neurons (as determined by the number of TH^+^ neurons) did not show a significant change in Otp-null ARC (Fig. 5d, e), suggesting that Otp is dispensable for the development of POMC- and GHRH-neurons. Consistent with the latter idea, the number of Dlx1^+^ neurons did not change significantly in Otp-null mice (Fig. 5e). These results also show that, in the developing ARC, Otp does not repress the expression of Dlx1/2, while Dlx1/2 inhibits Otp expression. Interestingly, despite the lack of Otp expression in KNDy-neurons (Supplementary Fig. 1a), Kiss1 expression was reduced in Otp-null mice at E18.5 (Supplementary Fig. 4a).

Taken together, our data highlight an essential role of Otp in directing the generation of AgRP-neurons.

### Dlx1/2 inhibits AgRP-neuronal fate via suppression of Otp

**Fig. 6:**
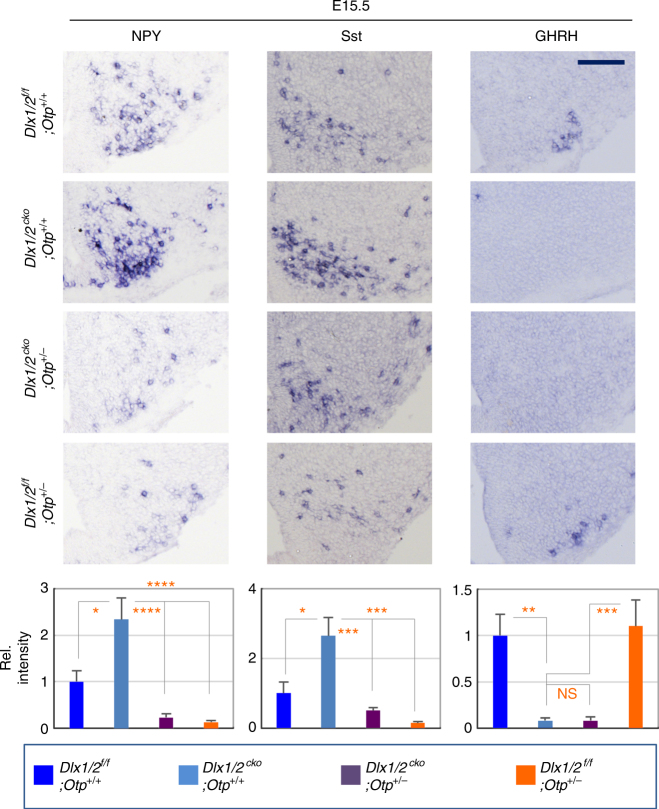
Dlx1/2 inhibits AgRP-neuronal fate via suppression of Otp. In ISH analyses, the expression of NPY and Sst in E15.5 *Dlx1/2*^*cKO*^*;Otp*^*+/*^^−^ embryos (*n* = 5) was significantly lower than that observed with their littermate *Dlx1/2*^*cKO*^*;Otp*^*+/+*^ embryos (*n* = 3) and rather comparable to that observed with *Dlx1/2* ^*f/f*^*;Otp*^*+/+*^ (*n* = 3) and *Dlx1/2* ^*f/f*^*;Otp*^*+/*^^−^ (*n* = 11) embryos. In contrast, the expression of GHRH was comparable between *Dlx1/2*^*cKO*^*;Otp*^*+/+*^ and *Dlx1/2*^*cKO*^*;Otp*^*+/*^^−^ embryos. Quantification was performed with multiple embryos as indicated and at least three sections from each embryo, and representative images for only one side of the ARC are as shown. Scale bars, 100 μm. Student’s *t*-test results are as indicated: ∗*p* < 0.05, ∗∗*p* < 0.01, ∗∗∗*p* < 0.001, and ∗∗∗∗*p* < 0.0001. Bars represent mean, error bars represent the SEM

The increased Otp expression in *Dlx1/2*^*cKO*^ ARC, combined with the critical role of Otp in AgRP-neuron formation, begs the question of whether the de-repressed Otp in the absence of Dlx1/2 drives the gain-of ectopic AgRP-neurons in *Dlx1/2*^*cKO*^ mice. If this is the case, the reduction of Otp levels in *Dlx1/2*^*cKO*^ mice will normalize AgRP-neuronal generation, but it will not restore GHRH-neuronal formation. To test this idea, we deleted a single copy of *Otp* in *Dlx1/2*^*cKO*^ mice (*Dlx1/2*^*cKO*^;*Otp*^*+/−*^) and monitored the development of AgRP- and GHRH-neurons. Remarkably, the aberrant gain-of AgRP-neurons in *Dlx1/2*^*cKO*^ mice was corrected when Otp levels were lowered in *Dlx1/2*^*cKO*^;*Otp*^*+/*^^−^ mice, but GHRH-neurons remained missing in *Dlx1/2*^*cKO*^;*Otp*^*+/*^^−^ mice (Fig. 6). These results establish that Dlx1/2 suppress AgRP-neuronal fate by inhibiting Otp expression during ARC development.

## Discussion

**Fig. 7:**
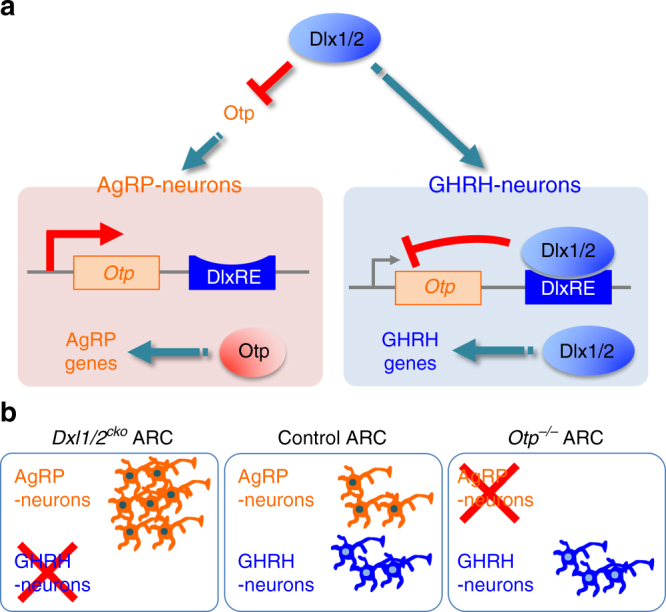
Our working models. **a** A balanced production of AgRP- and GHRH-neurons is directed at least in part by the Dlx1/2-Otp gene regulatory axis, by which Dlx1/2 suppress Otp expression in developing GHRH-neurons. It remains to be determined if a similar gene regulatory axis that functions to block developing AgRP-neurons from adopting a GHRH-neuronal fate also exists. Also unclear is how Dlx1/2 and Otp control GHRH- and AgRP-neuronal genes, respectively. **b** Schematic representation of dysregulated production of AgRP- and GHRH-neurons in *Dlx1/2*^*cKO*^ and *Otp*^*−/−*^ mice relative to control mice

In this paper, we present a novel gene regulatory axis consisting of the homeodomain transcription factors Dlx1/2 and Otp, which operates in the developing ARC. We propose that this Dlx1/2-Otp axis is critical for striking a balance between the embryonic generation of GHRH- and AgRP-neurons, and that this mechanism may contribute to a coordinate control of growth and energy homeostasis at postnatal stages (Fig. 7).

Given the interdependence between nutritional/energy status and linear growth^5–7^, it is possible that the development of energy homeostasis-controlling AgRP-/POMC-neurons and growth-promoting GHRH-neurons is synchronized, which may involve the differentiation of common precursors to related but distinct neuronal types. Our findings that the Dlx1/2-Otp gene regulatory axis is essential for the segregation of GHRH- and AgRP-neuronal fates indicate that the developmental pathways for GHRH- and AgRP-neurons are interconnected. In this regard, it is noteworthy that Npy-GFP^+^ cells can be divided into Otp^+^ and Otp^−^ populations. While a majority (~86%) of Npy-GFP^+^ cells express Otp in E14.5 ARC, ~14% of Npy-GFP^+^ cells do not (Fig. 5a). Interestingly, ~14% of Npy-GFP^+^ cells express Dlx1 in E14.5 ARC (Fig. 1d). Combined with the finding that Otp and Dlx1 expression is mutually exclusive in the embryonic ARC (Fig. 4i), these data raise the intriguing possibility that Npy-GFP^+^ population is composed of Otp^+^/Dlx1^−^ and Otp^−^/Dlx1^+^ cells, which give rise to AgRP- and GHRH-neurons, respectively. On a related note, in the developing ARC, POMC^+^ precursors generate several neuronal types, including POMC-, AgRP-, and fertility-regulating KNDy/Kiss1-neurons^4,34^. Future investigation into the ontogeny of arcuate neurons, such as the relationship among POMC^+^ and Npy-GFP^+^ precursors and their progeny, will reveal how different types of arcuate neurons are produced in the right ratio during development to perform highly interconnected functions at postnatal stages.

Our data suggest that Dlx1/2-mediated repression of the *Otp* gene is the key to consolidating the GHRH-neuronal identity and keeping the number of AgRP-neurons in check. In Dlx1/2-null ARC, the number of Otp^+^ AgRP-neurons appeared to be increased at the expense of Otp^−^ GHRH-neurons (Figs. 2 and 4j; Supplementary Fig. 3a). Given the total number of neurons did not change significantly in Dlx1/2-null ARC (Supplementary Fig. 3b), the presumptive GHRH-neurons might have adopted AgRP-neuronal fate in the absence of Dlx1/2. It is also possible that, in addition to GHRH-neurons, some non-GHRH-neuronal types that normally express Dlx1/2 (Fig. 1b) convert to AgRP-neurons in Dlx1/2-null ARC. The deletion of a single copy of the *Otp* gene in Dlx1/2-null mice was sufficient to prevent the formation of ectopic AgRP-neurons (Fig. 6). Combined with a complete loss-of-AgRP-neurons in Otp-null mice (Fig. 5d), these results suggest that Otp plays an instructive role in AgRP-neuronal fate specification and that Dlx1/2 inhibit the AgRP-neuronal fate mainly by repressing *Otp*, whose erroneous expression can divert the precursors to AgRP-neuronal fate. Notably, we and others have shown that Dlx1 and Otp exhibit a complementary expression pattern in multiple regions of the hypothalamus (Fig. 4i)^16–18^, pointing to the possible cross-repressive interactions between Dlx1 and Otp. Indeed, Otp was de-repressed in the ARC of *Dlx1/2*^*cKO*^ mice (Fig. 4j), but Dlx1 expression did not alter in Otp-null ARC (Fig. 5e), indicating that Dlx1/2 repress Otp, but not vice versa in the developing ARC. In contrast, in the PVN, the deletion of *Otp* resulted in the expansion of Dlx1 expression domain^16^. Thus, different repressive mechanisms may operate to generate the boundary between Dlx1/2- and Otp-expressing cells in a cell context-dependent manner.

AgRP-neurons are activated by food deprivation, and the stimulation of AgRP-neuronal circuitry promotes food intake and suppresses energy expenditure^1^. The recent single-cell transcriptome analyses in the adult ARC revealed that AgRP-neurons are divided into the two molecularly distinct subtypes, Sst-expressing AgRP-neurons (Sst^+^AgRP^+^) and Sst-negative AgRP-neurons (Gm8773^+^AgRP^+^)^3^. However, it remains unclear if Sst^+^AgRP^+^ and Gm8773^+^AgRP^+^ subtypes play distinct roles in controlling energy balance and apetite. In light of the finding of AgRP-neuronal subtypes, it is noteworthy that an AgRP-neuronal subtype expressing CRFR1 plays a regulatory role in adapting to cold stress^29^. When this CRFR1^+^ AgRP-neuronal subtype was hyper-activated by the deletion of *CRFR1*, the female mutant mice showed lower body temperature with no change in food intake under basal conditions and dropped body temperature significantly more rapidly than control mice when exposed to 5 °C, but the male mutant mice did not show body temperature changes^29^. These data suggest that CRFR1-expressing AgRP-neuronal subtype is involved in controlling body temperature, and needs to be inhibited to enable cold-induced thermogenesis. According to the gene expression profile studies in the adult ARC^3^, CRFR1^+^ AgRP-neurons are the Sst^+^AgRP^+^ subtype. Intriguingly, we found that *Dlx1/2*^*cKO*^ mice showed the increased levels of AgRP- and Sst-neurons in the ARC (Fig. 2). Similarly to the condition that hyper-activates CRFR1^+^ AgRP-neuronal subtype^29^, *Dlx1/2*^*cKO*^ female mice exhibited significantly lower body temperature and were more susceptible to the cold stress than control mice without a change in food intake (Fig. 3e, h; Supplementary Fig. 3d), suggesting that the activity of CRFR1-expressing Sst^+^AgRP^+^ subtype is augmented in *Dlx1/2*^*cKO*^ mice. The increased cold-susceptibility in female *Dlx1/2*^*cKO*^ mice may be responsible, at least partly, for the higher death rate of female mice (Fig. 3a), particularly given that the standard temperature of our animal room (22 °C) could provide constant cold stress to mice^35,36^. *Dlx1/2*^*cKO*^ mice also showed a reduced energy expenditure as monitored by oxygen consumption and carbon dioxide production (Fig. 3f), as well as a significantly decreased locomotor activity (Fig. 3g), both of which were likely reflected in a late-onset obesity (Fig. 3d). Taken together, in *Dlx1/2*^*cKO*^ mice, only an AgRP-neuronal subtype suppressing the energy consumption, but not the subtype stimulating the feeding behavior, may have been selectively increased. Alternatively, it is also possible that both AgRP-neuronal subtypes were aberrantly gained in *Dlx1/2*^*cKO*^ mice, but only a specific subtype (i.e., CFRF1^+^/Sst^+^ AgrP-neurons) was able to form the functional neuronal circuitry. A better understanding of AgRP-neuronal subtypes at the molecular, connectivity, physiology, and functional levels will help resolve this issue. Yet another possibility for future investigation is a possibility of GHRH-neurons interplaying with AgRP-neurons in food intake control. Although, GHRH-null mice showed enhanced food intake^37^, it is possible that the entire loss-of-GHRH-neurons themselves (which could be the case with our *Dlx1/2*^*cKO*^ mice) may differently alter the feeding behavior, particularly given the inverse correlation between GH/GHRH levels and the circulating level of the anorexic hormone leptin^5–7^.

Our finding that *Dlx1/2*^*cKO*^ mice show dwarfism, an absence of GHRH-neurons, and dysregulated GH signaling (Fig. 3b, c) establish Dlx1/2 as essential players in the gene network directing the development of GHRH-neurons. Notably, the deletion of *Otp* in Dlx1/2-null mice failed to recover GHRH-neurons although it rescued the aberrant gain-of AgRP-neurons (Fig. 6), indicating that the Dlx1/2-Otp axis is not involved in promoting the GHRH-neuronal fate. Instead, in driving GHRH-neuron development, Dlx1/2 may act as an upstream activator of Hmx2/3 and Gsx1^24,25^, given the marked downregulation of Hmx2 and Gsx1 in Dlx1/2-null ARC (Supplementary Fig. 3c). Hmx2/3 are needed for the expression of Gsx1^24^, which directly binds and triggers the transcription of the *GHRH* gene^25^. Together, these findings propose the gene regulatory cascade, Dlx1/2-Hmx2/3-Gsx1-GHRH, which promotes the GHRH-neuronal identity in the developing ARC. Considering our results that Dlx1/2 can function as either transcriptional activator or transcriptional repressor depending on a gene context in the same cell type (Fig. 4f, Supplementary Fig. 7a), it is interesting to postulate that Dlx1 triggers the Dlx1/2-Hmx2/3-Gsx-GHRH cascade via its transcriptional activator function, while repressing Otp as a transcriptional repressor in segregating the fates for GHRH- and AgRP-neurons in the precursors (Fig. 7). The future investigation is needed to understand the molecular basis for the gene context-specific dual roles of Dlx1/2.

## Methods

### Animals

All mice were housed in a pathogen-free animal facility under a normal 12 h light, 12 h dark cycle with ad libitum access to normal chow and water, unless otherwise noted. All studies were approved by the Institutional Animal Care and Use Committee of Oregon Health and Science University. *Npy-hrGfp* (#006417), *Pomc-eGfp* (#009593) and *Nkx2.-Cre* (#008661) mice were purchased from the Jackson Laboratory. *Otp*^*+/*^^−^ and *Dlx1/2*^*f/f*^ mice were kindly provided by Dr. Dario Acampora and Dr. Magdalena Petryniak, respectively.

### Body temperature measurements

Anesthetized mice were implanted with Implantable Programmable Temperature and Identification Transponder (IPTT-300; Bio Medic Data Systems). Body temperature was measured using DAS-7009 reader (Bio Medic Data Systems).

### Metabolic studies and food intake

Oxygen consumption and carbon dioxide production were measured using the indirect calorimetry system Oxymax (Columbus instruments). Data were collected after 72 h of acclimation with singly housed mice. Whole-body composition of 3–4-month-old mice were analyzed with EchoMRI^TM^ system. Intake of food was analyzed by weighing the amount of food in each cage at 6 am and 6 pm for two consecutive days. The mice were weighed on the second day and the food intake was analyzed per mouse and per g of body weight. For measuring locomotor activity, circadian home cage activity was continuously measured using a home cage sensor system and mLog software (BioBServe, Germany). Mice were singly housed and given food and water ad libitum in cages placed on a conventional Metro rack. Data were acquired every second and were averaged into 30 min bins for analysis.

### Chick in ovo electroporation

The P29 ChIPseq peak region and Dlx1 were subcloned into CMV-GFP reporter and RCAS expression vectors, respectively. Chicken eggs were incubated in a humidified chamber for 48–52 h. DNA constructs were injected into the lumen of chick embryonic spinal cord at HH stage 11–13. Electroporation was performed using a square wave electroporator (BTX). Incubated chicks were collected and fixed in 4% paraformaldehyde 3 days after electroporation.

### ChIP

E16/E16.5 WT mouse hypothalami were dissected out and homogenized before being cross-linked with 1% formaldehyde for 10 min at room temperature, followed by quenching with 125 mM glycine. The cells were washed in PBS, and lysed in cell lysis buffer (5 mM PIPES, pH 8, 85 mM KCl, 0.5% NP40) and nuclear lysis buffer (50 mM Tris-HCl, pH 8, 10 mM EDTA, 1% SDS, protease inhibitor), followed by sonication. Cell lysates were redissolved in dilution buffer (0.5% Triton X-100, 5 mM EDTA, 150 mM NaCl, 25 mM Tris-HCl, pH 7.5, 0.5% deoxycholate, 0.1% SDS, protease inhibitor cocktail) and incubated with IgG and protein A agarose beads for 1 h for immunoclearing. The supernatant, collected after quick spin-down, was immunoprecipitated by our home-made anti-Dlx1 antibody or control IgG (Santa Cruz) overnight, followed by incubation with protein A agarose for 2 h at 4 °C. The beads were sequentially washed with RIPA (0.1% SDS, 1% NP40, 1 mM EDTA, 50 mM Tris-HCl, pH 8.0, 150 mM NaCl, 0.5% deoxycholate), high salt buffer (same as RIPA except 500 mM NaCl) and LiCl buffer (0.25 M LiCl, 1% NP40, 0.5% deoxycholate, 1 mM EDTA, 150 mM Tris-HCl, pH 8.0) for 10 min at each step. The beads were subsequently washed with TE buffer (10 mM Tris-HCl, 1 mM EDTA) three times. The protein/chromatin complexes were eluted in elution buffer (1% SDS, 1 mM EDTA, 0.1 M NaHCO_3_, 50 mM Tris-HCl, pH 8.0) and reverse cross-linked by incubating at 65 °C overnight, followed by incubation at 50 °C for <2 h with proteinase K. The DNA was extracted with phenol/chloroform, followed by ethanol precipitation and solubilization in water.

### Immunohistochemistry and in situ hybridization

Anesthetized mice were perfused transcardially with PBS and then with 4% paraformaldehyde. Brains were removed and placed in 4% paraformaldehyde overnight, washed with PBS, and incubated with 30% sucrose. Embryos were fixed in 4% paraformaldehyde and incubated in 30% sucrose. Brain sections (12 μm thick) were prepared with a cryostat and incubated with primary antibodies at 4 °C overnight and followed by 1–2 h incubation with fluorescence-conjugated secondary antibodies (1:500, Jackson Immuno Research). To cover the representative ARC areas, we used at least three sections that are located ~144–168 μm from each other. For ISH, antisense RNA probes were labeled with digoxigenin-UTP (Roche Diagnostics) according to the manufacturer’s protocol. Hybridization was performed at 68 °C overnight. Hybridized sections were washed in 4× SSC and 0.2× SSC solution, and incubated with anti-digoxigenin-AP antibody (11093274910, Roche Diagnostics; 1:4000) overnight. The sections were subjected to color reaction with NBP/BCIP. The VECTASTAIN Elite ABC Kit (PK-6101, Vector Labs) was used according to the manufacturer’s instruction for immunohistochemistry assay following ISH. The probes for αMSH, AgRP, NPY, Sst, GHRH, Gad1, and Dlx1 were described previously^38^. The antibodies used for immunohistochemistry are anti-GR (SC-1004, Santa Cruz; 1:500), anti-Isl1 (home-made; 1:3000), our home-made anti-Dlx1 (antibody), our home-made anti-OTP (using the 183–325 aa region of mouse Otp as the antigen), anti-Bsx^33^ (1:2000), rabbit anti-NeuN (Ab177487, Abcam; 1:2000), rabbit anti-TH (AB152, Millipore; 1:500), and anti-GFP (GFP-1020, aves labs; 1:2000) antibodies.

### Image analysis and quantification

A length of 12 μm brain sections covering the entire arcuate nucleus were placed onto a series of slides with 4–5 sections on each slide. The distance between sections in single slide is 144–168 μm. One slide from each mouse that contains matched sections was used to compare controls and mutants. Zeiss Axio imager 2 with apotome was used to image in situ hybridization and immunohistochemistry result. Integrated density measurement in Image J software was used to analyze densitometry. For cell counting, analyze particles measurement in image J was used to count specifically immuno-stained cells in the arcuate nucleus.

### Co-immunoprecipitation and luciferase assay

Dlx1 and Dlx2 WT or Dlx2-QE mutant were subcloned into pcDNA3 plasmid with N-terminal triple Flag tag. ChIPseq peak P27 and P29 regions were cloned into TK-luciferase vector and used for luciferase assays. HEK293 cells were maintained in DMEM supplemented with 10% fetal bovine serum and penicillin/streptomycin. For co-immunoprecipitation assay, cells were seeded into 10 cm dishes and transiently transfected with either Dlx1 or Dlx2 expression vector using the standard calcium phosphate method. Anti-Flag M2 antibody (F3165, Sigma) was used for immunoprecipitation (1 μg) and immunoblotting (1:3000), and a full size image with size markers is presented in supplementary Fig. 5. For luciferase assays, cells were seeded into 48-well plates and transiently transfected with luciferase reporter and Dlx1/2-expression vectors using SuperFect (Qiagen) according to the manufacturer’s instruction. The actin-β-galactosidase plasmid was co-transfected for normalization of the luciferase results. Data are shown in relative luciferase units (mean ± s.d.).

### RNA extraction and quantitative RT-PCR analysis

Total RNAs were extracted from a piece of mouse liver using Trizol (Invitrogen) and reverse-transcribed using Thermo Scientific Maxima H Minus Reverse Transcriptase. The following primers were used with SYBR Green kit (Thermo Scientific Luminaris Color Higreen, #K0371) for quantitative RT-PCR of IGF1: 5′-TCATGTCGTCTTCACACCTCT-3′ and 5′-TCCACAATGCCTGTCTGAGG-3′.

### Statistical analyses

Statistical differences were determined by Student’s *t*-test. Statistical significance is displayed as follows: ∗*p* < 0.05, ∗∗*p* < 0.01, ∗∗∗*p* < 0.001, and ∗∗∗∗*p* < 0.0001. The *p*-values for the CO_2_/VO_2_ measurements, as well as the body temperature measurement in the cold room were also analyzed using two-way ANOVA test. The home cage activity and food intake during the light and dark periods were analyzed separately using repeated-measures ANOVA with genotype as the between-subjects factor.

### Data availability

Dlx1 ChIPseq data that support the findings of this study have been deposited in the National Center for Biotechnology Information Gene Expression Omnibus (GEO) and are accessible through the GEO Series accession number GSE104372. All other relevant data are available from the corresponding author on request.

## Electronic supplementary material

Supplementary Information

Description of Additional Supplementary Files

Supplementary Data 1
