# Supplementary material for: Dlx1/2 and Otp coordinate the production of hypothalamic GHRH- and AgRP-neurons
Source: Nat Commun. 2018 May 23;9:2026. doi: 10.1038/s41467-018-04377-4 (PMC5966420; doi:10.1038/s41467-018-04377-4)
Supplement: Supplementary file 1 — Supplementary Information [file 41467_2018_4377_MOESM1_ESM.pdf]

Supplementary information

Dlx1/2 and Otp coordinate the production of hypothalamic GHRH- and AgRP-neurons

Lee et al.

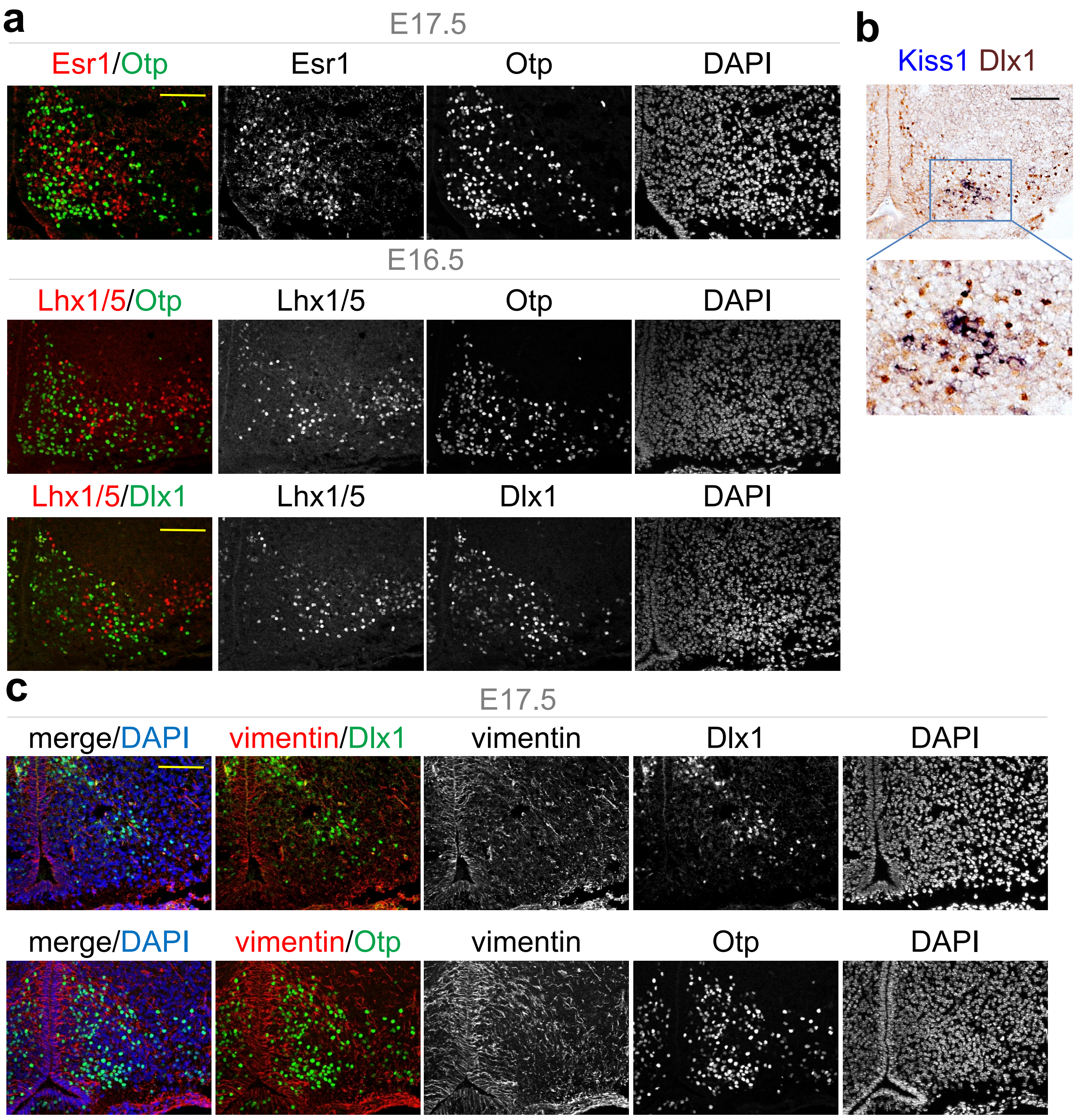

Supplementary Fig. 1. Dlx1 and Otp are not expressed in KNDy-neurons and tanycytes in the ARC. (**a**) IHC analyses of Dlx1, Otp, Esr1, and Lhx1/5 in E16.5 and E17.5 WT embryos. Esr1 marks KNDy- and POMC-neurons, and Lhx5 marks KNDy-neurons[1]. Dlx1 and Otp are not coexpressed with both markers. (**b**) IHC for Dlx1 combined with ISH for Kiss1, the marker for KNDy-neurons. Kiss1 and Dlx1 are not coexpressed in the same neurons in the ARC. (**c**) Vimentin is a tanycyte marker, and IHC for vimentin, Dlx1 and Otp indicates that Dlx1 and Otp are not expressed in vimentin^{+} tanycytes. The scale bar is for 100 μm.

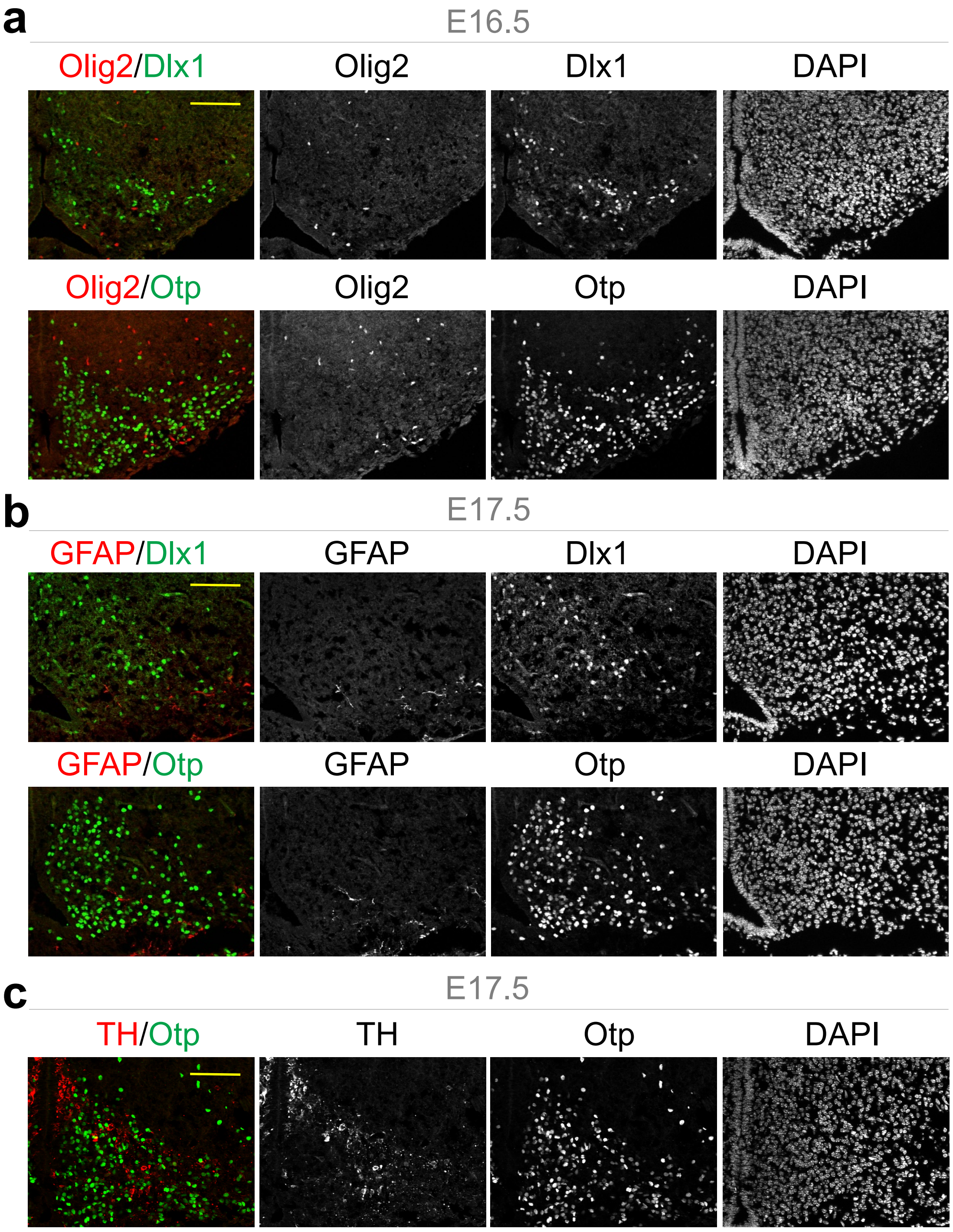

Supplementary Fig. 2. Our IHC analyses revealed that Dlx1 and Otp are expressed in neither Olig2^{+} oligodendrocytes (**a**) nor GFAP^{+} astrocytes (**b**), and that Otp is not expressed in TH^{+} neurons. The scale bar is for 100 μm.

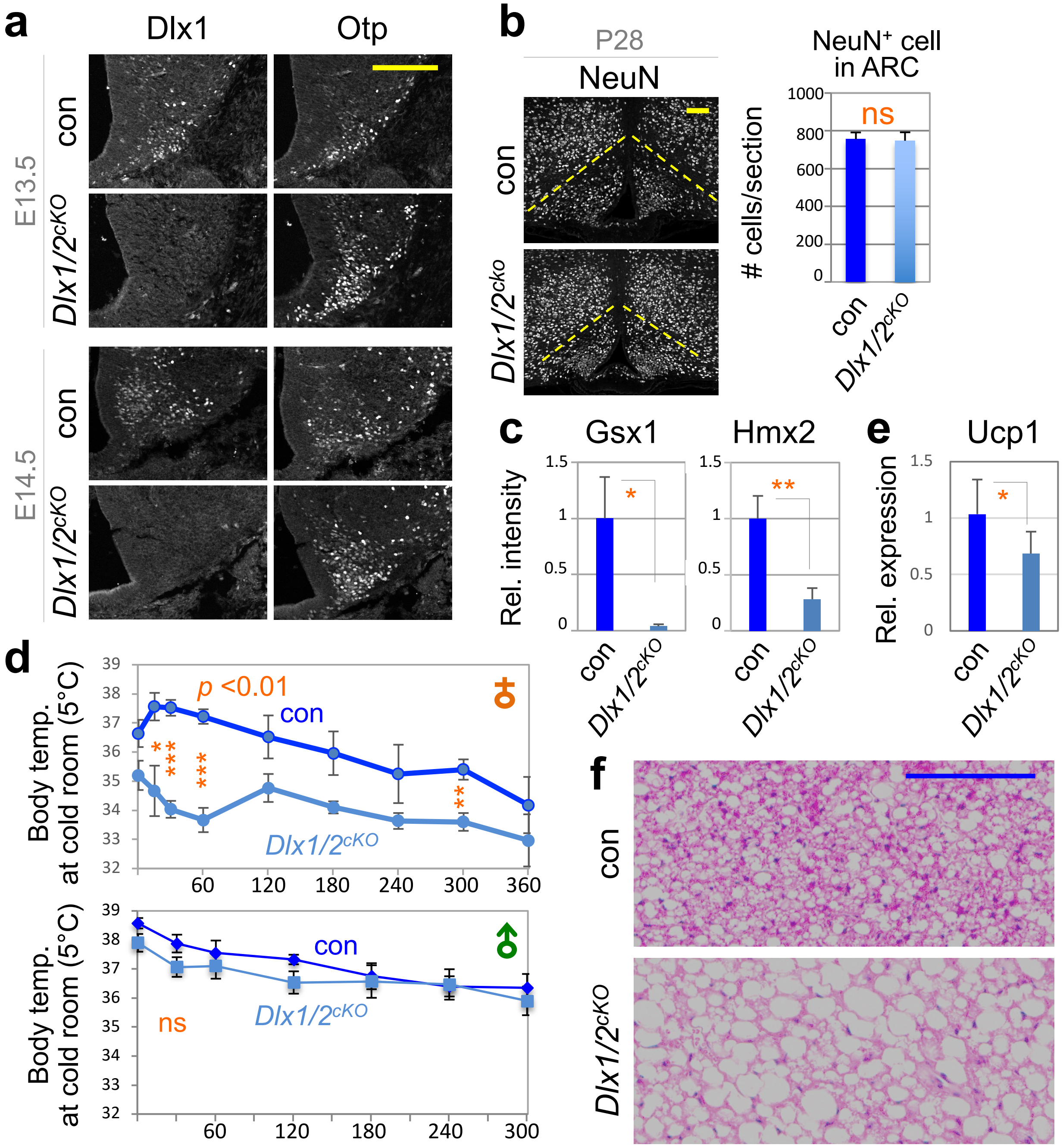

Supplementary Fig. 3. (**a**) IHC analyses of Dlx1 and Otp in *Dlx1/2*^{cKO} mice and their controls at E13.5 and E14.5 using our home-made antibodies against Dlx1 and Otp. (**b**) IHC analyses of the number of neurons in the ARC of *Dlx1/2*^{cKO} mice (n=3) and their controls (n=3) at P28 using anti-NeuN antibody. Of note, the size of the ARC (outlined in yellow lines) is smaller in *Dlx1/2*^{cKO} mice relative to their controls. (**c**) ISH signals for Gsx1 and Hmx2 are significantly reduced in the ARC of *Dlx1/2*^{cKO} (n=4) relative to controls (n=4) at E15.5. (**d**) Body temperature measurement upon cold challenge (in group cages, given *Dlx1/2*^{cKO} mice were severely ill and died in single cages) revealed that female *Dlx1/2*^{cKO} mice (n=3) lose body temperature more rapidly than their littermate female control mice (n=4) for the first 60 min. In contrast, male *Dlx1/2*^{cKO} mice (n=3) did not show any difference from their littermate male control mice (n=4). *P* values in two way ANOVA are as indicated. Some time points for female mice also showed statistically significant differences in Student's t test, as indicated ($^{*}p < 0.05$, $^{**}p < 0.01$, $^{***}p < 0.001$). (**e, f**) Our qRT-PCR and H&E staining revealed that, after 60 min of cold exposure, the BATs of female *Dlx1/2*^{cKO} mice (n=4) showed significantly reduced expression of Ucp1 (**e**) as well as hypertrophy of brown adipocytes (**f**) relative to control female BATs (n=6). The scale bars, 100 μm. All error bars represent the SEM.

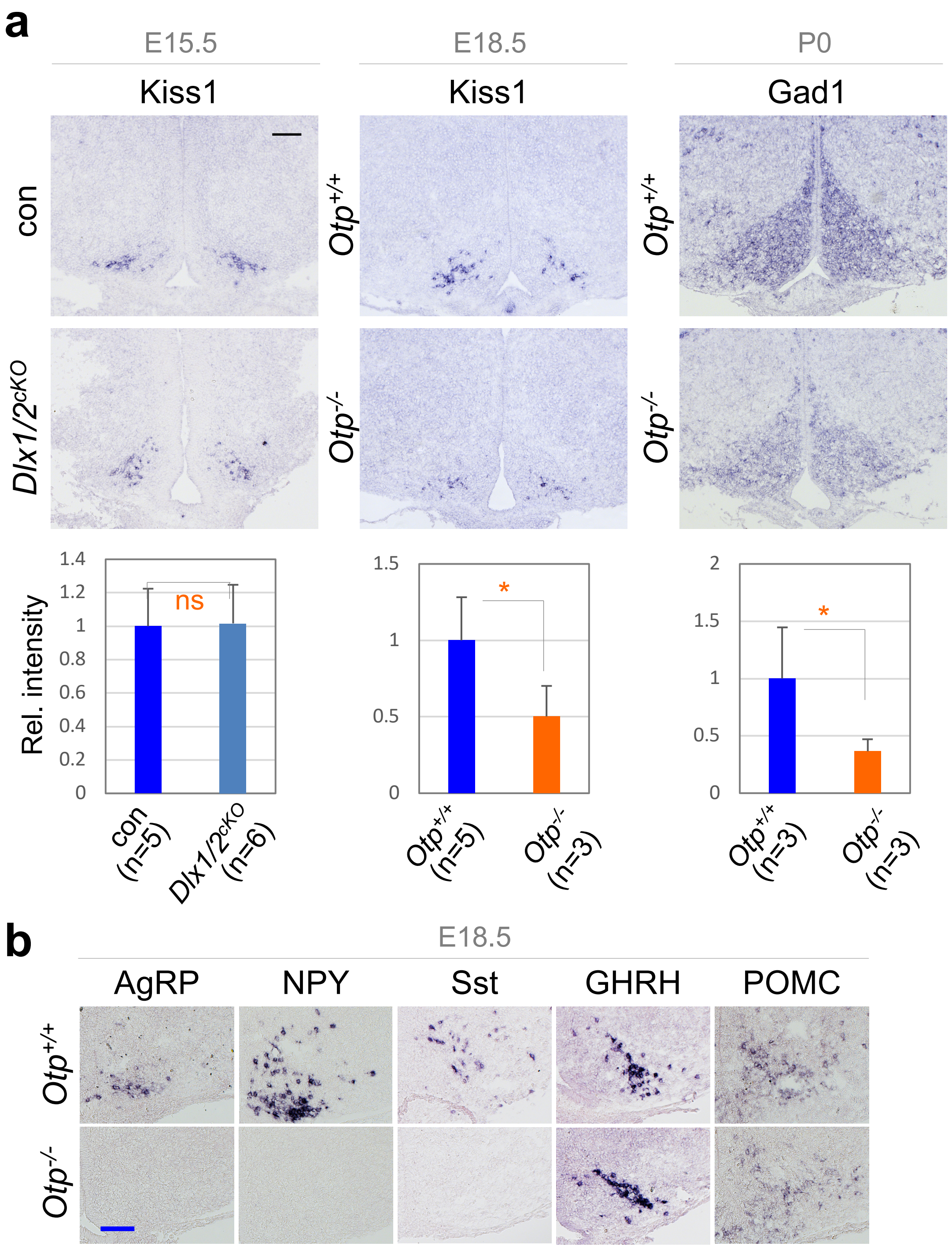

Supplementary Fig. 4. (**a**) ISH analyses of E15.5 *Dlx1/2*^{cKO} and E18.5 Otp-null embryo for Kiss1 as well as P0 Otp-null embryo for Gad1 revealed no changes in Kiss1 expression in Dlx1/2-null embryos and reduced expression of Kiss1 in Otp-null embryos. Gad1 expression was also reduced in P0 Otp-null embryos, which is likely due to the loss of AgRP-neurons that are mostly GABAergic. Error bars represent the SEM. (**b**) ISH analyses of E18.5 Otp-null embryo (only one analyzed) revealed reduced expression of AgRP, NPY and Sst but comparable expression of GHRH and POMC in comparison to their littermate control embryo (n=1). We also obtained similar results with P0 Otp-null embryos (n=3) relative to their littermate control embryos (n=4). Images for only one side of the ARC are shown. The scale bar is for 100 µm (**a**,**b**).

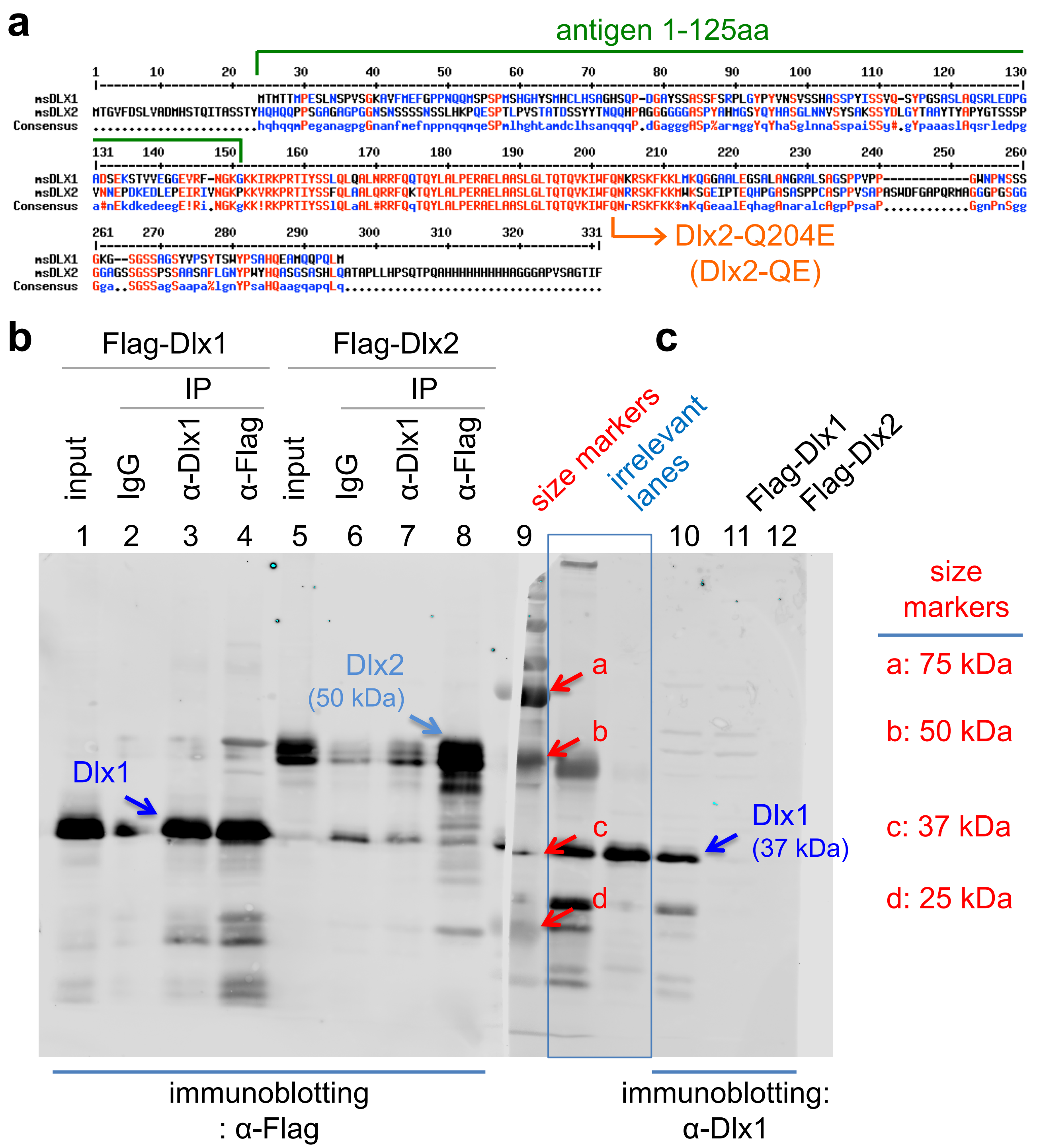

Supplementary Fig. 5. (**a**) Sequence alignment of Dlx1 and Dlx2. The Dlx1 antigenic region that we used to raise the antibodies as well as the non-DNA-binding mutant form of Dlx2, Dlx2-Q204E, are as indicated. (**b,c**) Dlx1-specificify of our antibody in immunoprecipitation (**b**) and immunoblotting (**c**). In both experiments, our Dlx1 antibody worked only with Dlx1 but not with Dlx2. For these experiments, HEK293 cells were transiently transfected with the expression vectors for Dlx1 and Dlx2, which are fused to three copies of Flag epitopes at the N-terminus. All samples (including two irrelevant lanes as indicated) were loaded into a single gel (#1-12), and divided into two blots, as evidenced by a split of the size marker lane (#9).

a

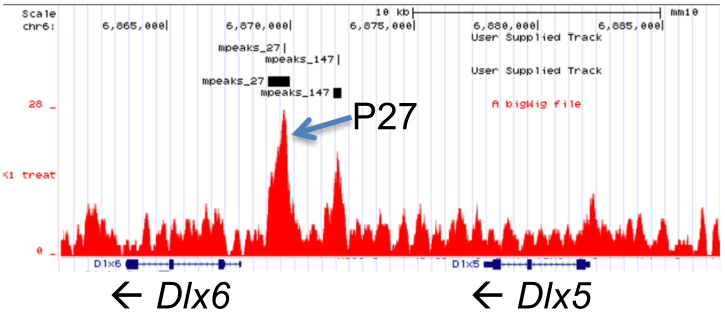

b

```
mouse     TGTTTTTAATCTGCATGGTGGACCTCGTATTTTAATTTTTGGGATAAAGAAAAAGCT-CAGATCCC
rat       CGTTTTTAATCTGCATGGCGGACCTTGTGTTTTAATTTTTGGGATAAAGAAAAAGCTCAGCTCAG-
human     TGTTTTTAATTTATATTGTGGAACTTACATTTAAATTTTTAGGATAAAGAAAAAACT---TAGCCC
pig       CGTTTTTAATTTGTGTTGTGGACTTTACATATTAATTTTTAGGATAAAGAAAAAACTTAG---CCC
dog       TGTTTTTAATGTGTGCTGTGGACTTTATATTTCAATTTTTAGGATAAAGAAAAAACT---TAACCC
cow       T--TTTTAATTTGTGTTGTGGACTTCACGTATGCATTTTTAGGGTAAAGAAAAAACTTCA---CCC
horse     T--TTTTAATCTGTGTTGTGGACTTTACATGTTAATTTTTACGGTAGAGAAAAAACT---TAGACC
chick     TGTTTTTAATGTGTGCTGTGGACTTTATATTTCAATTTTTAGGATAAAGAAAAAACT---TAACCC
conserved    ******* *     * ***   *     * *   ******   * ** ******* **

mouse     --ACAGATGTGTACCCTACTAATGACAGTAGTTAACCTGTCAGCTGAAAGAATGTAACCTTCCTTT
rat       --ACAGATGTGTACC---CTAATGACGGTAGTTAGCCTGTCAGTTGAAAGAATGTAACCTTCCCTT
human     ACAAATATGTGTATTCTAGTAATGGCAGTAGTTAACCTGTCAGTTGAAATAATGTAACCTTCCTTT
pig       ACAAAGATGTGTCTTCTAGTAATGGCAGTTGTTAACCTGTCAGTTGAAAGAATGTAACCTTCCTTT
dog       ACAAAGATGTGTCTTCTAGTAATGGCAGTAGTTAACCTGTCAGTTGAAAGAATGTAACCTTCCTTT
cow       ACAAAGATGTGTCTTCTAGTAATGGCAGTAGTTAACCTGTCAGTTGAAAGAAGGTAACCTTCCTTT
horse     CCAAAGATGTGTCTTTTAGTAATGGCAATAGTTAACCTGTCAGTTGAAAGAATGTAACCTTCCTTT
chick     ACAAAGATGTGTCTTCTAGTAATGGCAGTAGTTAACCTGTCAGTTGAAAGAATGTAACCTTCCTTT
conserved   * * ******    *  ***** ** ****** ******** ***** ** ********** **

mouse     TCTCCCTCTCA-GACATAAAGATAATCTCATCTGAGGGCATTTATGTGAAGAAATAACAGCATGGC
rat       TCTCCCTCTCA-GACATAAAGATAATCTCATCTGAGGGCATTTGTGTGAAGAAATAACAACATGGC
human     TCTCCTTTTCATGACATAAAGATAATCTCATCTGAGGGCATTTATGTGAAGAAATAACAGCATGGC
pig       TCTCCTTTTCATGACATAAAGGTAATCTCATCTGAGGGCATTTATGTGAAGAAATAACAGCATGGT
dog       TCTCCTTTTCATGACATAAAGGTAATCTCATCTGAGGGCATTTATGTGAAGAAATAACAGCATGGC
cow       TCTCCTTTTCATGACATAAAGATAATCTCATCTGAGGGCATTTATGTGAAGAAATAACAGCATGGC
horse     TCTCCTTTTCATGACATAAAGGTAATCTCATCTAAGGGCATTTATGTGAAGAAGTAACAGCATGGC
chick     TCTCCTTTTCATGACATAAAGGTAATCTCATCTGAGGGCATTTATGTGAAGAAATAACAGCATGGC
conserved ***** * *** ********* *********** ********* ********* ***********

mouse     ATGGGGAAGCTACTGGCTCATTAATCCTGGCATCTACTTCCTACAGGGCGCCTTGATTAGTTAG
rat       ATGGTGGAACTACTGGCTCATTAATCCTGGCATCTACTTCCTACAGGGC-TCTTGATTAGCTAG
human     ATGGTGAAACCACTGGCTCATTAATCCTGGCATCTACTTCCTACAGGGTTCCGTGATTAGTTAG
pig       ATGGTGAAACTACTGGCTCATTAATCCTGGCATCTACTTCCTACAGGGTTCCTTGATTAGTTAG
dog       ATGGTGAAACTACTGGCTCATTAATCCTGGCATCTACTTCCTACAGGGTTCCTTGATTAGTTAG
cow       ATGGTGAAACTACTGGCTCATTAATCCTGGCATCTACTTCCTACAGGGTTCCTTGATTAGTTAG
horse     ATGGTGGAACTACTGGCTCATTAATCCTGGCATCTACTTCCTACAGGGTTCCTTGATTAGTTAG
chick     ATGGTGAAACTACTGGCTCATTAATCCTGGCATCTACTTCCTACAGGGTTCCTTGATTAGTTAG
conserved ****** *****************************************   *************
```

Supplementary Fig. 6. (**a**) Our Dlx1 ChIPseq peaks included a peak encompassing the previously reported binding site for Dlx1/2 in the intergenic region of *Dlx5* and *Dlx6* (arrow, Ref[2]). (**b**) The evolutionarily highly conserved A/T-rich motifs, which may serve as the potential direct binding sites for Dlx1/2, are found within the peak P29 in *Otp*. Overall, the sequences in this intergenic P29 region are highly conserved (highlighted in green), supporting the vital role of P29 in Otp expression.

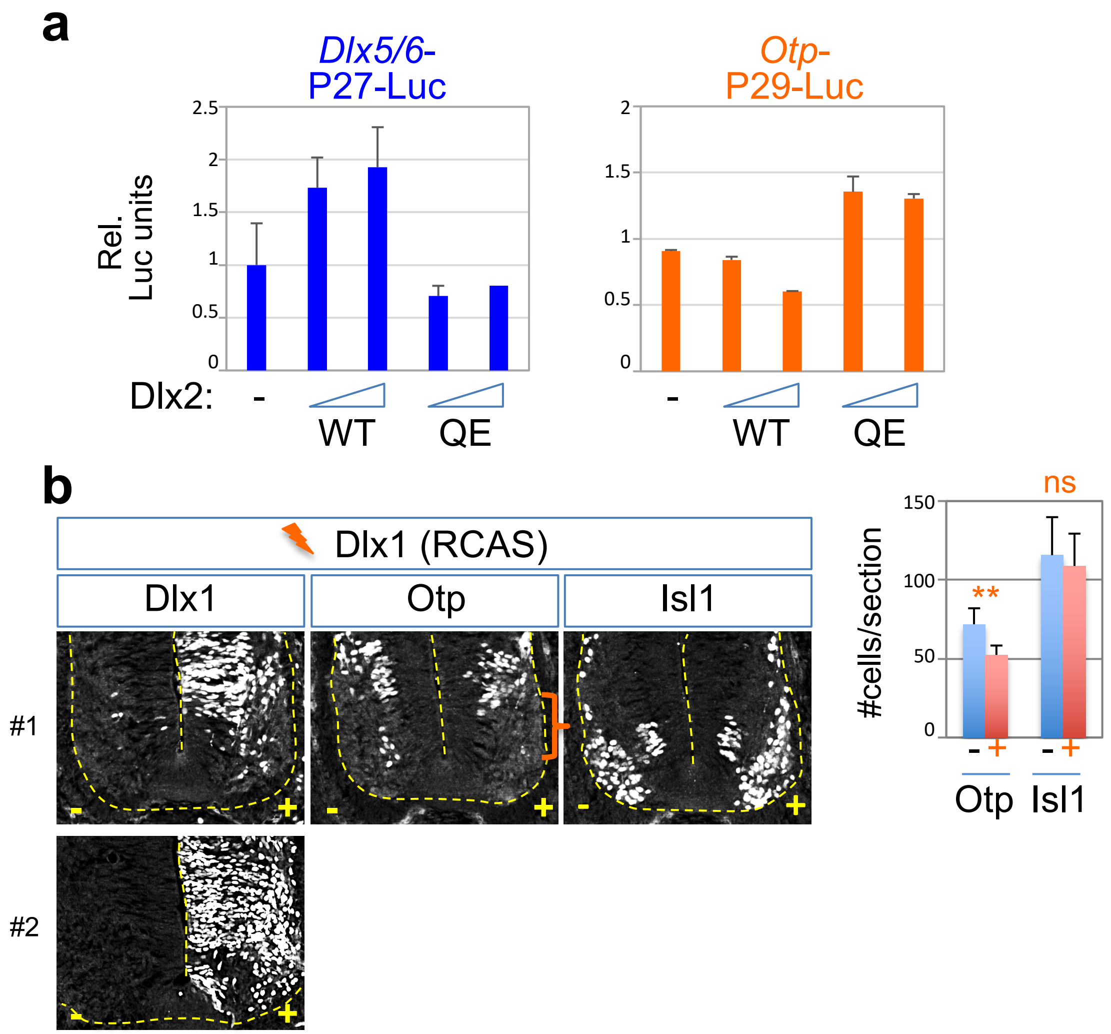

Supplementary Fig. 7. (**a**) Luciferase reporter assays to assess the transcriptional response of P29 and P27 by WT Dlx2 and a DNA-binding defective mutant form of Dlx2 (QE). The reporter assays were repeated four times, which produced similar results. A representative set of results is as shown. Error bars represent the SD. (**b**) In ovo electroporation of Dlx1-expression vector significantly suppressed the ventral expression of Otp (bracket), while it did not change the expression pattern of Isl1, as demonstrated by quantification of the number of Otp^+ and $Isl1^+$ cells (n=5). Notably, as shown in #2 embryo, Dlx1 was not expressed in the developing spinal cord, unlike Otp, and several $Dlx1^+$ cells in the unelectroporated side of #1 embryo represent a secondary infection by the viral expression vector RCAS, which was used to express Dlx1 in these experiments. +, electroporated side; -, unelectroporated side. Error bars represent the SEM.

Supplementary references

1. Campbell, J. N. *et al.* A molecular census of arcuate hypothalamus and median eminence cell types. *Nature neuroscience* **20**, 484-496, doi:10.1038/nn.4495 (2017).
2. Zerucha, T. *et al.* A highly conserved enhancer in the Dlx5/Dlx6 intergenic region is the site of cross-regulatory interactions between Dlx genes in the embryonic forebrain. *The Journal of neuroscience : the official journal of the Society for Neuroscience* **20**, 709-721 (2000).
