# Supplementary material for: Dlx1/2 and Otp coordinate the production of hypothalamic GHRH- and AgRP-neurons
Source: Nat Commun. 2018 May 23;9:2026. doi: 10.1038/s41467-018-04377-4 (PMC5966420; doi:10.1038/s41467-018-04377-4)
Supplement: Supplementary file 2 — Description of Additional Supplementary Files [file 41467_2018_4377_MOESM2_ESM.pdf]

Description of Additional Supplementary Files

File Name: Supplementary Data 1
Description: Dlx1 ChIPseq peaks and their associated genes
